# Novel Natural Glycyrrhetinic Acid-Derived Super Metal Gel and Its Highly Selective Dyes Removal

**DOI:** 10.3390/gels8030188

**Published:** 2022-03-19

**Authors:** Shengzhu Guo, Kaize Su, Huiji Yang, Wende Zheng, Zhen Zhang, Song Ang, Kun Zhang, Panpan Wu

**Affiliations:** 1 School of Biotechnology and Health Sciences, Wuyi University, Jiangmen 529020, China; shengzhuguo@163.com (S.G.); skz3176933515@163.com (K.S.); 15875045599@163.com (W.Z.); z1833918@163.com (Z.Z.); 2 International Healthcare Innovation Institute Jiangmen, Jiangmen 529040, China; 3 College of Chemistry, Beijing Normal University, Beijing 100875, China; 201911150911@mail.bnu.edu.cn

**Keywords:** glycyrrhetinic acid-derived, gel, Cu^2+^-triggered shrinkage, antimicrobial activity, dyes removal

## Abstract

Hydrogels play important roles in function materials, especially in wastewater treatment, that could solve the problems of microbial infections and dye pollutions. Herein, a natural glycyrrhetinic acid-derived gel was successfully constructed by forming hierarchical assemblies of the glycyrrhetinic acid derivatives (GA-O-09) with Cu^2+^. Interestingly, the GA-O-09/Cu^2+^ gel exhibited Cu^2+^-triggered shrinkage, which was helpful for spontaneous self-demolding through the shrinkage process with a precise amount of Cu^2+^. Moreover, the gel showed excellent antimicrobial activity against *Staphylococcus aureus* and methicillin-resistant *Staphylococcus aureus* (MRSA) with minimum inhibitory concentrations (MICs) at 2.5 μg/mL and 5.0 μg/mL, respectively. Furthermore, the resultant GA-O-09/Cu^2+^ gel showed an excellent performance in dyes removal; the adsorption capacity at equilibrium (q_e_) could reach 82.91 mg/g according to a pseudo-second-order model, and it was better than most reported dye adsorbent materials. The experimental result suggested that the electrostatic interactions of the hydrogel with the cationic dyes and the hydrogel swelling were responsible for the possible dye removal mechanism of GA-O-09/Cu^2+^ gel. Therefore, our study holds the promise of a better future, for such a hydrogel could be used as an antibacterial and dye removal material.

## 1. Introduction

During the development of industry, the process of wastewater with dyes has been a spiny problem. Dye pollutions and bacterial infections, [1] which are the main problems in water resources, can cause deleterious effects on human health. [2] Therefore, developing novel efficient wastewater treatment materials that could help in dyes removal and bacterial inhibition is extremely urgent and has attracted great attention in the field of healthcare. Hydrogel is a promising material for dye adsorption and antibacterial application because of its high hydrophilicity and unique three-dimensional network [3,4]. Hence, constructing novel hydrogels is a significant strategy to solve the problem of wastewater treatment.

Taking full advantage of natural products in biomedical applications is a current hot topic [5,6]. It has been reported that glycyrrhetinic acid derivatives could be used as basis functional materials for supramolecular hydrogels and that they exhibited excellent bioactivity [7]. In our former work, we found that one of the natural glycyrrhetinic acid derivatives, GA-O-09, could form hydrogels and exhibit excellent antibacterial activity [8,9]. However, GA-based hydrogels have been the object of many studies in other applications besides wastewater treatment. Thus, we are motivated to develop a novel hydrogel that could be used in wastewater treatment, one that shows a performance in both dye absorbance and antibacterial activity [10,11].

Herein, a natural glycyrrhetinic acid-derived gel was successfully constructed by forming hierarchical assemblies of GA-O-09 with Cu^2+^. Interestingly, the super metal hydrogel showed that the ratio on shrinkage reached a high value of 54.7 ± 1.2%. Most importantly, the shrunken gel showed an excellent performance in bacteriostatic activity, and its MIC was 2.5 μg/mL. Meanwhile, the Cu^2+^-triggered shrunken gel exhibited a superior performance in the removal of dye molecules when compared to most reported hydrogels [12]. For example, methylene blue (MB) could be removed by the gel after 3 h, which was more efficient than other adsorbents.

## 2. Results and Discussion

### 2.1. Preparation of GA-O-09/Cu^2+^ Hydrogel

Due to the strong coordination between metal ions and pyridine groups, the assembled property of Cu^2+^ with GA-O-09 was explored in the mixture solvents of water and ethanol by using the ‘stable to inversion of a test tube’ method [9]. Interestingly, the metal hydrogel showed a performance in shrunken gel, which started to shrink after its formation at room temperature.

**Figure 1:**
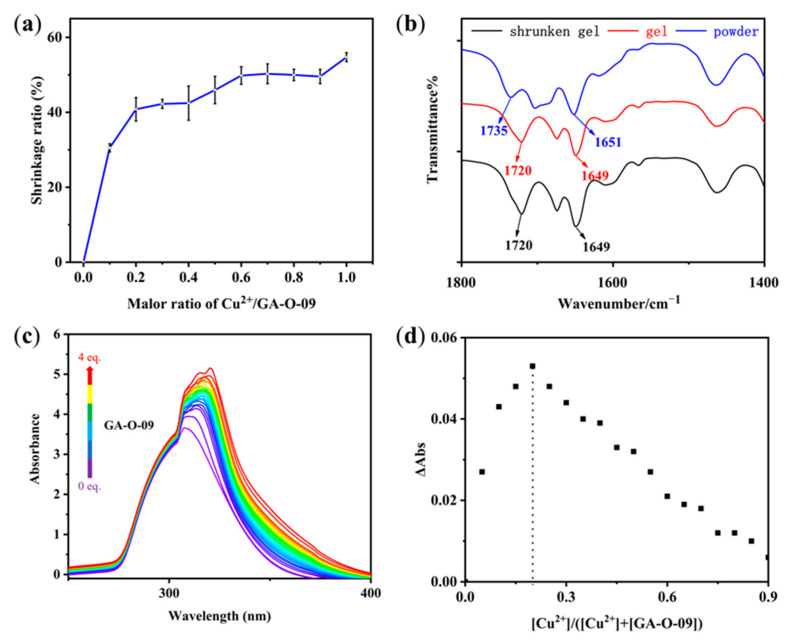
(**a**) Shrinkage ratio of the GA-O-09/Cu^2+^ gel changed with the molar ratios of Cu^2+^ and GA-O-09. (**b**) FT-IR spectra of GA-O-09 powder, GA-O-09 gel, and GA-O-09/Cu^2+^ shrunken gel. (**c**) UV-vis titration of Cu^2+^ with GA-O-09. CH_3_OH/CHCl_3_ (*v*/*v*, 1/2) was used as the solvent to preclude the formation of precipitates or gel during the titration. The concentration of Cu^2+^ was at 7 mmol/L, and the concentration of GA-O-09 ranged from 0 to 28 mmol/L. (**d**) Job’s plot of UV-vis titration at 320 nm showing 1:4 complex formations between Cu^2+^ and GA-O-09.

GA-O-09 gels containing different equivalents of Cu^2+^ were prepared to further evaluate their shrinkage performance. The shrinkage phenomenon was more remarkable with an increasing amount of Cu^2+^, more solvents were extruded from the gel, and the shrinkage ratio (Figure 1a) continued to rise. The shrinkage ratio reached 30.54 ± 0.86% with the addition of the 0.1 equivalent of Cu^2+^ and reached a high value of 54.71 ± 1.2% in the presence of an equivalent of Cu^2+^, which was much higher than that of other reported shrinkable gels triggered by metal ions [13].

### 2.2. The Possible Assembly Mechanism

The possible assembly mechanism of the shrinkable gel was explored in our study. As shown in the FT-IR experiments in Figure 1b, the C=O stretching vibrations from the carbonyl bond moved from 1735 and 1651 cm^−1^ to 1720 and 1649 cm^−1^, respectively, which indicated that the hydrogen bonds formed through the oxygen atoms of the carbonyl groups in the aggregated GA-O-09 gel with water molecules. Moreover, the FT-IR spectra showed no prominent difference between GA-O-09 gel and GA-O-09/Cu^2+^ gel, which revealed the unperturbed hydrogen bonds in the shrunken gel, which proved that Cu^2+^ was coordinated with the pyridine groups instead of the carbonyl bonds. Meanwhile, UV–vis titration was conducted to obtain binding ratios where the absorption peak of Cu^2+^ shifted from 324 nm to 353 nm with the addition of GA-O-09 (Figure 1c). In order to study the binding stoichiometry between Cu^2+^ and GA-O-09, a job’s plot was employed to confirm this, as shown in Figure 1d. Then, the molar ratio of Cu^2+^ to GA-O-09 was gradually increased from 0 to 1; meanwhile, the intensity of the absorption at 320 nm was recorded with the changes of the molar ratio. Therefore, we can obtain the absorption intensity of all the molar ratios, and one of the emissions reached a maximum at a ratio of about 0.25 in the job’s plot, which confirmed the 1:4 stoichiometry between Cu^2+^ and GA-O-09 with the formation of tetrapyridinecopper (II) [14,15].

**Figure 2:**
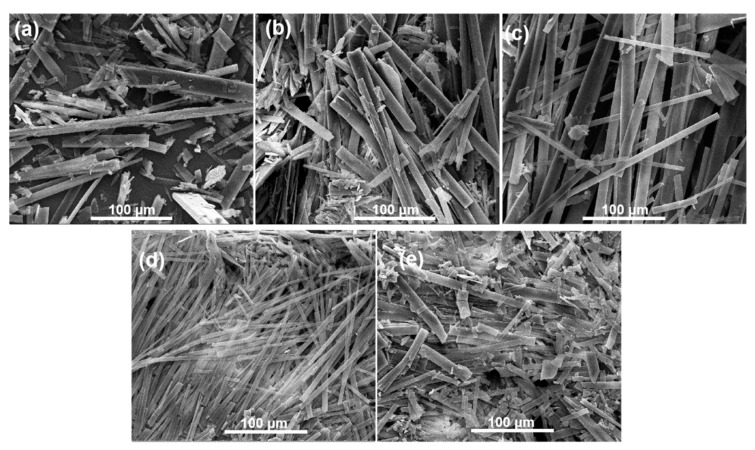
SEM images of GA-O-09 gel with different equivalents of copper ions: (**a**) 0 equivalent; (**b**) 0.2 equivalent; (**c**) 0.5 equivalent; (**d**) 0.8 equivalent; (**e**) 1.0 equivalent.

Besides, scanning electron microscopy (SEM) observations were conducted to investigate the morphological transformation of the GA-O-09/Cu^2+^ gel upon shrinkage. In the SEM images (Figure 2 and Figure S1), the GA-O-09 gel existed as a sparse, orderly rod structure, while the GA-O-09/Cu^2+^ gel appeared as a compact one, which was responsible for the macroscopic shrinkage of the GA-O-09/Cu^2+^ gel and indicated that coordination interaction was one of the main driving forces for the gelation.

**Figure 3:**
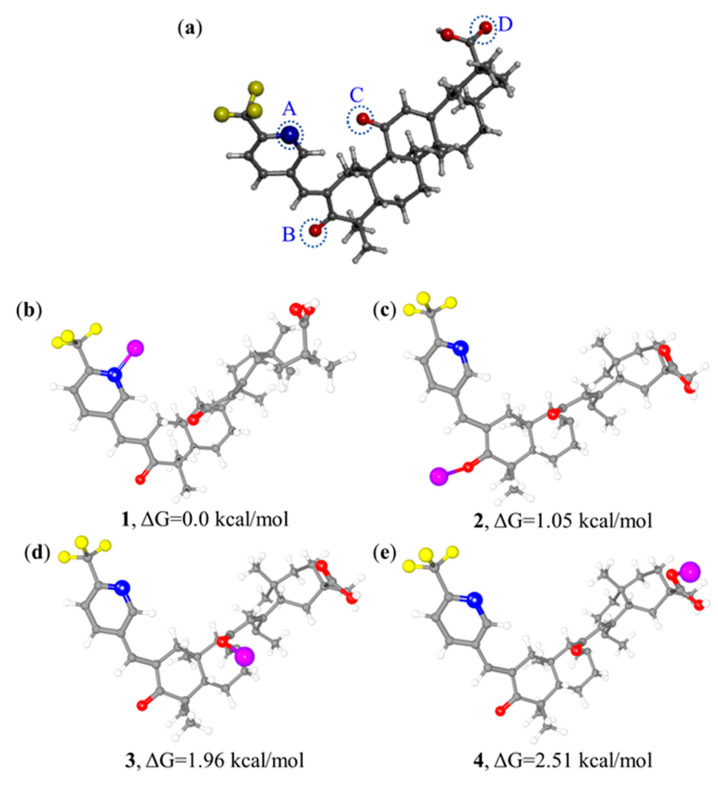
(**a**) Crystal structure of GA-O-09 with four potential binding sites: A, B, C and D. (**b**–**e**) Optimized structures of [GA-O-09·Cu^2+^] complexes with copper ion binding at the potential sites, respectively, and corresponding Gibbs free energy values of the four optimized structures. Carbon atoms are presented in gray, hydrogen atoms in white, oxygen atoms in red, nitrogen atoms in blue, and copper atoms in purple.

In addition, density functional theory (DFT) calculations of [GA-O-09·Cu^2+^] complexes were performed at the B3LYP/6-31G* level in theory [16]. The calculation of the Gibbs free energy for the feasible structures of the [GA-O-09·Cu^2+^] complex with four binding sites for the copper ion suggested that **1** was more stable by 1.05, 1.96, and 2.51 kcal·mol^−1^ when compared with **2**, **3**, and **4**, respectively, as shown in Figure 3. Apparently, the coordination between the pyridine group and copper ion could be achieved more easily and stably than that between the carbonyl and copper ion.

**Figure 4:**
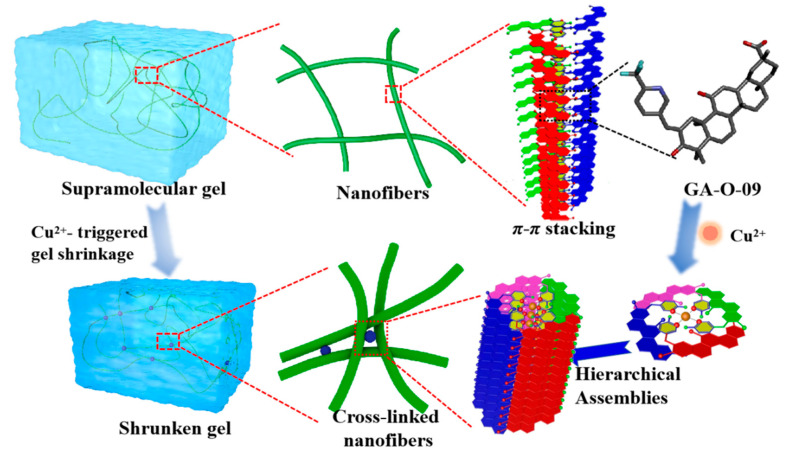
The schematic representation of the Cu^2+^-triggered macroscopic gel shrinkage with changes at the nanoscale.

All in all, the shrinkage mechanism was proposed as shown in Figure 4. Firstly, the supramolecular assemblies were formed by the GA-O-09 gelators in an orderly rod structure by π-π stacking of the pyridine group and the hydrophobic interaction of the glycyrrhetinic acid skeleton. Secondly, the copper ion served as a bridge in the adjacent rod structure, which resulted in the formation of a network of cross-linked rod structures, leading to the gelling solvents being extruded and to the gel volume declining, which was ultimately observed as a macroscopic shrinkage [17].

### 2.3. The Antimicrobial Activity Assays

The antimicrobial activity of the gel against gram-positive bacteria was evaluated and is shown in Table S1, indicating that such a hydrogel had the potential to be used for antimicrobial materials [18,19,20]. It was found that the gels with different concentrations of Cu^2+^ showed the same antimicrobial activity against *Staphylococcus aureus* with the minimum inhibitory concentration (MIC) at 2.5 μg/mL, which meant that the copper ion did not affect the antimicrobial activity. Besides, it was ascertained that such a hydrogel had a potential antibacterial activity with the MIC at 5 μg/mL against MRSA, a major nosocomial pathogen that causes wildly severe morbidity and mortality, as a result of which the application of this antibacterial hydrogel would show brilliant promise in the treatment of multidrug resistance in the future. This hydrogel had no effect against gram-negative; however, it could be used as an antibacterial hydrogel, since it showed a performance against *Staphylococcus aureus* and MRSA. 

### 2.4. Selective Removal of Dyes in Aqueous Solution

**Figure 5:**
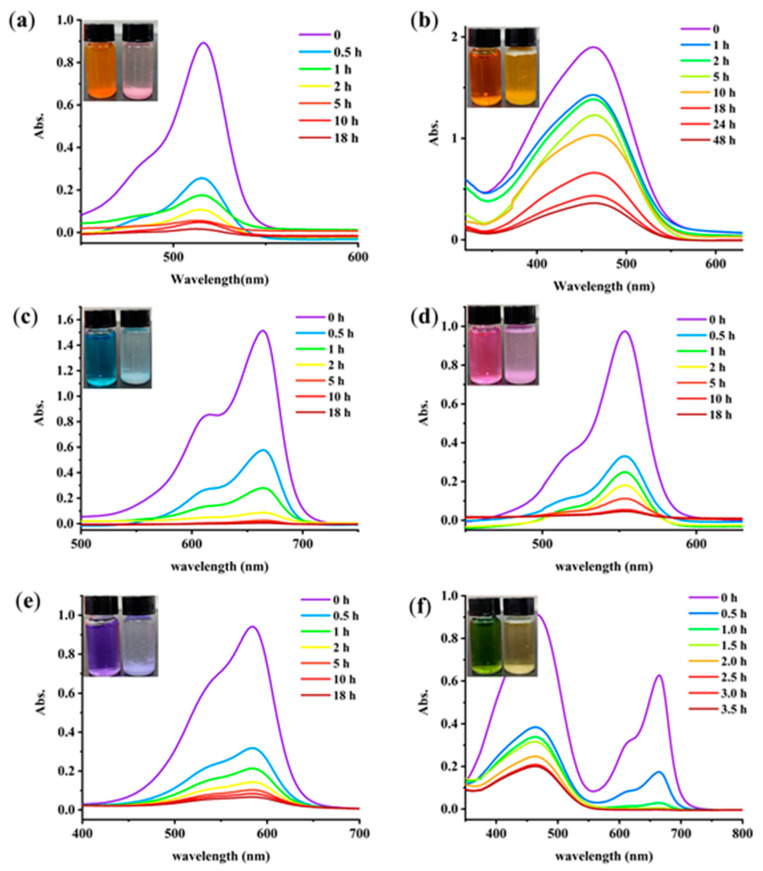
UV–vis spectra of the dye solutions suspended with the GA-O-09/Cu^2+^ shrunken gel: (**a**) EY, (**b**) MO, (**c**) MB, (**d**) R6G, (**e**) MC and (**f**) a mixture of MO/MB for the indicated time, (inset) photos of the dye solutions before (left) and after (right) the removal by the GA-O-09/Cu^2+^ shrunken gel.

Toxic dye-based industrial effluents are hazardous to the ecological balance and environment. Therefore, seeking convenient and efficient adsorbents for the decontamination of wastewater is a very attractive project [21]. UV−vis spectroscopy was applied to monitor the effect of dyes removal. The chemical structures of dyes are shown in Figure S2, including anionic dyes eosin Y (EY), methyl orange (MO), and cationic dyes such as rhodamine 6G (R6G), methylene blue (MB), and methylrosanilnium chloride (MC). As shown in Figure 5, the GA-O-09/Cu^2+^ xerogel was suspended in an aqueous solution of the pollutants (250 mg/L, 5.00 mL) at ambient temperature for 18 h. With the exception of methyl orange, all the dyes showed remarkable decolorization. Overall, it may be said that such a GA-O-09/Cu^2+^ gel can be used in dye removal materials. Besides, MB was completely removed within two hours by the gel when it was mixed with MO. Moreover, the GA-O-09/Cu^2+^ gel showed better abilities than the GA-O-09 gel (Figure S3).

**Figure 6:**
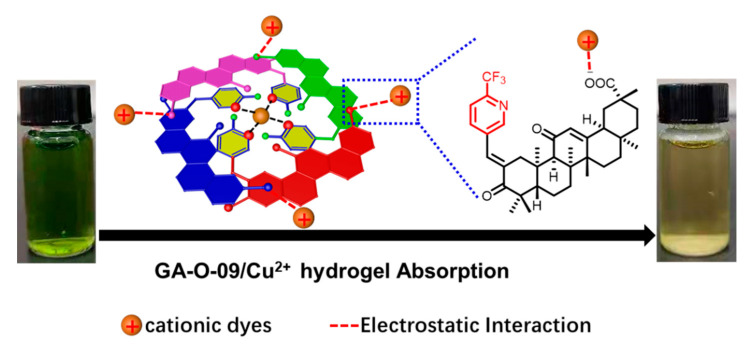
The possible mechanism of GA-O-09/Cu^2+^ gel for cationic dyes removal.

Besides, the differences observed in trials performed with the different types of dyes and the dye removal mechanism could be both explained by the BET and Zeta potential test. Different types of dyes show different sizes and charge densities. The BET surface area of the GA-O-09/Cu^2+^ hydrogel was 8 m²/g, and the Zeta (Figure S4) potential of the GA-O-09/Cu^2+^ hydrogel was negative, indicating that the surfaces of the gel were negatively charged, which was helpful to cationic dyes removal by electrostatic interactions. Meanwhile, the FT-IR (Figure S5) and SEM (Figure S6) provided evidences that cationic dyes interacted with hydrogels. Therefore, the possible mechanism of GA-O-09/Cu^2+^ gel for cationic dyes removal (Figure 6) was attributed predominantly to electrostatic interactions of the hydrogel.

**Figure 7:**
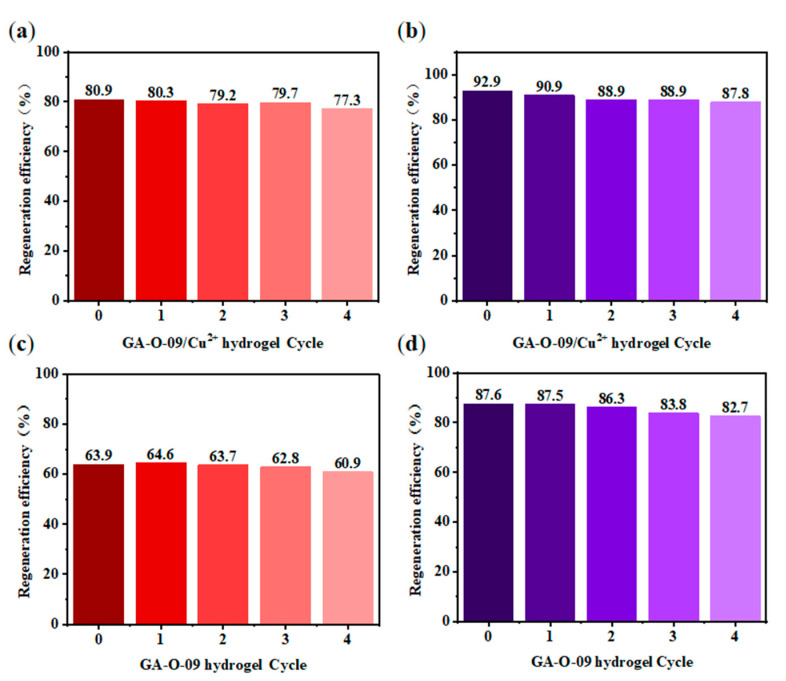
Regeneration cycles of (**a**,**c**) GA-O-09/Cu^2+^ hydrogel and (**b**,**d**) GA-O-09 hydrogel after the adsorption of MO (red) and MC (purple) by washing with ethyl acetate at 25 °C, respectively.

**Figure 8:**
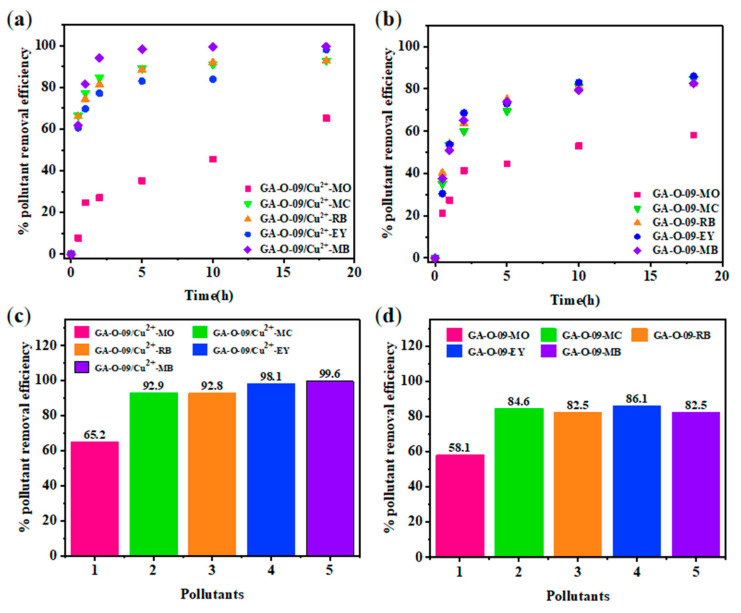
Adsorption process of each pollutant (250 mg/L) at different points in time: (**a**) GA-O-09/Cu^2+^ hydrogel; (**b**) GA-O-09 hydrogel; and removal efficiency of each pollutant at the equilibrium state: (**c**) GA-O-09/Cu^2+^ hydrogel; (**d**) GA-O-09^2+^ hydrogel.

For adsorbent materials, reusability is one of the most important properties [22]. After the contaminants were absorbed by the GA-O-09/Cu^2+^ xerogel, the corresponding GA-O-09/Cu^2+^ xerogel was filtered from the aqueous solution, and the water was purified. Then, the GA-O-09/Cu^2+^ xerogel was regenerated by soaking it in ethanol solvent and heated it to 45 °C for about 10 mins. Subsequently, after filtration and vacuum-drying at 70 °C, the GA-O-09/Cu^2+^ xerogel was recovered. In Figure 7, the reusability was performed via four repeated cycle tests. As a result, the adsorption property of the GA-O-09/Cu^2+^ xerogel demonstrated a tiny loss. Most importantly, the SEM (Figure S7) and FT-IR (Figure S8) of the regenerated GA-O-09/Cu^2+^ xerogel showed that the gel kept up the structure and confirmed its good stability. In addition, corresponding plots of the dyes removal efficiency as a function of time and net percentages of the pollutants removal efficiency were constructed and are shown in Figure 8. According to these results, the GA-O-09/Cu^2+^ xerogel showed a high pollutants removal efficiency of up to 98.1%.

**Figure 9:**
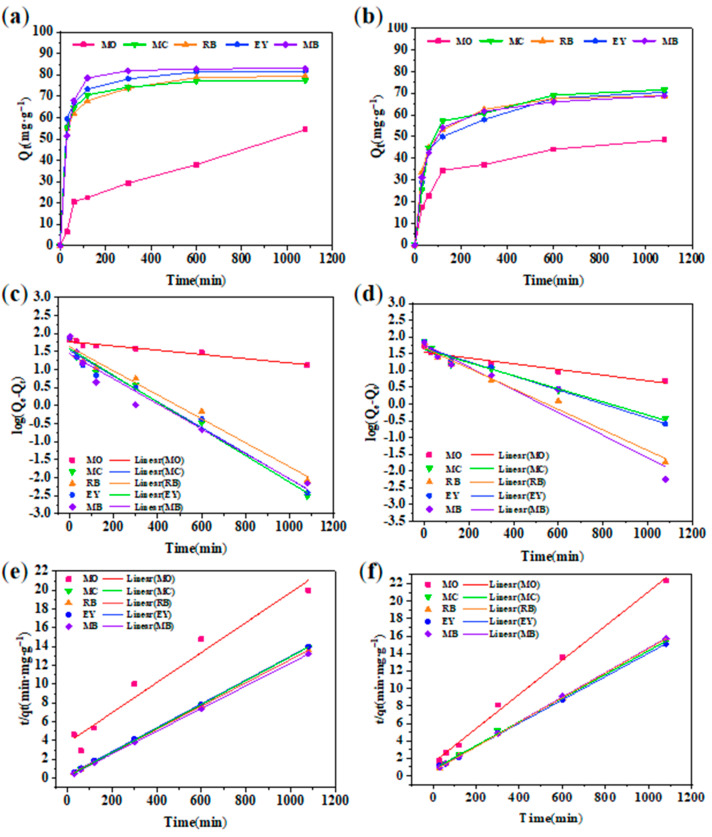
(**a**) Real-time monitoring of dyes absorption by GA-O-09/Cu^2+^ hydrogel; (**b**) pseudo-first-order kinetic plots of GA-O-09/Cu^2+^ hydrogel; (**c**) pseudo-second-order kinetic plots of GA-O-09/Cu^2+^ hydrogel; (**d**) Real-time monitoring of dyes absorption by GA-O-09 hydrogel; (**e**) pseudo-first-order kinetic plots of GA-O-09 hydrogel; (**f**) pseudo-second-order kinetic plots of GA-O-09 hydrogel.

Moreover, the kinetics of the adsorption process [23] were studied by two commonly used models. Figure 9 shows the adsorption capacities of GA-O-09 and GA-O-09/Cu^2+^ hydrogels for different dyes and their pseudo-first-order and pseudo-second-order linear regressions, respectively. The values of the adsorption rate constant (k), correlation coefficient (R^2^), and the adsorption capacity at equilibrium (q_e_) for all pollutants are listed in Table 1. It was found that the adsorption performance of the xerogel on the dye was more in line with the pseudo-second-order model, that its corresponding R^2^ was very close to 1, and that the theoretical equilibrium adsorption amount calculated by the formula was the actual equilibrium. In addition, it was also found that although the fitting effect of the pseudo-first-order model was not as good as that of the pseudo-second-order model, its R^2^ value was also greater than 0.9, indicating that physical adsorption was one of the indispensable factors. Meanwhile, the calculated maximum adsorbed quantity q_e_ of MB was 82.9 mg/g, which was much higher than that of the other adsorbents (Table 2). Therefore, the GA-O-09/Cu^2+^ hydrogel can be an effective adsorbent for organic dyes such as MB.

Besides, thermodynamic studies [22] were performed and are shown in Table 3 to study the influence of the temperature on the adsorption process of dye onto GA-O-09/Cu^2+^ hydrogel. It could be found that the obtained values of the Gibbs free energy change ΔG^0^ were negative and increased with the temperature, which indicated that the adsorption of dye molecules onto the GA-O-09/Cu^2+^ hydrogel was spontaneous in nature and became less favorable with an increasing temperature. Moreover, the negative values of enthalpy (ΔH^0^) and entropy (ΔS^0^) confirmed the exothermic nature of this adsorption process, as revealed by the Tempkin isotherm analysis.

## 3. Conclusions

In summary, a Cu^2+^-triggered shrinkage of a natural glycyrrhetinic acid-derived supramolecular gel with dyes removal and antimicrobial activity was successfully constructed. A shrinkage ratio of 54.71 ± 1.2% was achieved in the presence of one equivalent of Cu^2+^, which was much higher than that of other reported shrinkable gels triggered by metal ions. Besides, this GA-O-09/Cu^2+^ gel showed a superior performance in the removal of the dye molecule, with the calculated maximum adsorbed quantity q_e_ of MB having a high value of 82.91 mg/g, which was better than that of the other adsorbents. Furthermore, the GA-O-09/Cu^2+^ gel showed an excellent performance in terms of antimicrobial activity against *Staphylococcus aureus* and MRSA with MICs at 2.5 μg/mL and 5.0 μg/mL, respectively. This study implied that the GA-O-09/Cu^2+^ hydrogel showed promise as a potential material in wastewater treatment, showing both an antibacterial activity and dye removal ability.

## 4. Materials and Methods

All reagents were purchased from commercial suppliers of Adamas Reagent Ltd. (Shanghai, China), all of the other reagents were of analytical grade, and the water used in this work was of ultrapure grade.

Preparation of gel: The GA-O-09/Cu^2+^ hydrogel was prepared by cooling a hot solution of GA-O-09 and copper sulfate in ethanol/water (0.52/0.48, *v*/*v*) through coordination interactions.

Fourier transform infrared (FT-IR) spectra were obtained using a Bruker spectrometer. Scanning electron microscopy (SEM) images were acquired using an SU-8010 instrument (the accelerating voltage was 10 kV). Samples were freeze-dried before measurement. UV–vis titration was recorded using a TU-1901 Spectrometer (1.0 cm quartz cuvette). GA-O-09 (70 mM) and copper nitrate (7 mM) were dissolved in CH_3_OH/CHCl_3_ (*v*/*v*, 1/2) to preclude the formation of precipitates or gel during the titration. 

Dye adsorption experiment: 15 mg of xerogel were soaked in the dye solvent, shaken and mixed for 5 min, and then allowed to stand. Then, the suspension in the bottle (1.00 mL) was taken by a syringe at different intervals and then filtered immediately by using a LABMAX 0.2 μm membrane filter. UV–vis spectroscopy was used to determine the residual concentration of the pollutants in each sample.

## Figures and Tables

**Table 1:** Rate constants of two different kinetic models for hydrogels during adsorption of dyes with different charges.

Sample	Pseudo-First-Order Model			Pseudo-Second-Order Model		
	K_1_	q_e_	R^2^	K_2_	q_e_	R^2^
	(min^−1^)	(mg·g^−1^)		(mg·g^−1^·min^−0.5^)	(mg·g^−1^)	
GA-O-09-MO	0.00259	60.9074	0.8977	0.00026	50.9944	0.9968
GA-O-09-MC	0.00737	74.8911	0.9816	0.00025	73.5835	0.9987
GA-O-09-RB	0.00621	70.7946	0.9825	0.00038	71.1744	0.9999
GA-O-09-EY	0.00914	77.5211	0.9594	0.00027	74.7943	0.9992
GA-O-09-MB	0.00351	72.4886	0.9269	0.00034	71.1238	0.9999
GA-O-09/Cu^2+^-MO	0.00135	58.9521	0.9590	0.00007	78.4314	0.9601
GA-O-09/Cu^2+^-MC	0.00820	71.1574	0.9693	0.00095	80.8407	0.9999
GA-O-09/Cu^2+^-RB	0.00764	72.4269	0.9746	0.00062	80.8407	0.9998
GA-O-09/Cu^2+^-EY	0.00852	75.9784	0.9789	0.00053	78.8644	0.9999
GA-O-09/Cu^2+^-MB	0.00799	70.5791	0.9436	0.00084	82.9177	0.9999

**Table 2:** Comparison of adsorption capabilities of GA-O-09/Cu^2+^ hydrogel at room temperature with other adsorbents for MB.

Adsorbent	q_max_ (mg/g)	Ref.
MCM-41	74	[12]
TiO_2_/MCM-41	54	[22]
Reed biochar—hydroxyapatite	21	[24]
SnO_2_ quantum dots decorated silica nanoparticles	73	[25]
Co_3_O_4_/SiO_2_ nanocomposite	54	[26]
GA-O-09/Cu^2+^ hydrogel	83	**This work**

**Table 3:** Thermodynamic parameters calculated for the removal of dye by the GA-O-09/Cu^2+^ and GA-O-09 hydrogels (C_0_ = 250 mg/L, dosage = 3 g/L).

Thermodynamic Parameters							
Sample	ΔG^0^ (kJ·mol^−1^) at Different T (K)					ΔS^0^ (J·mol^−1^·K^−1^)	ΔH^0^ (kJ·mol^−1^)
	298	308	318	328	338		
GA-O-09-MO	−867.3	−867.3	−866.9	−866.1	−864.8	−0.0602	−885.6
GA-O-09-MC	−3644	−3642	−3639	−3636	−3631	−0.3141	−3739
GA-O-09-RB	−4724	−4722	−4720	−4718	−4709	−0.3533	−4831
GA-O-09-EY	−7045	−7030	−7027	−7069	−6988	−0.7008	−7255
GA-O-09-MB	−6965	−6961	−6960	−6957	−6956	−0.2109	−7027
GA-O-09/Cu^2+^-MO	−172.9	−171.6	−170.6	−169.1	−168.8	−0.1063	−204.4
GA-O-09/Cu^2+^-MC	−1496	−1486	−1481	−1480	−1477	−0.4502	−1627
GA-O-09/Cu^2+^-RB	−1126	−1104	−1089	−1084	−1082	−0.4960	−1261
GA-O-09/Cu^2+^-EY	−1768	−1758	−1754	−1752	−1748	−0.4443	−1897
GA-O-09/Cu^2+^-MB	−1125	−1115	−1111	−1109	−1106	−0.4542	−1257

## Data Availability

The study did not report any data.

## Supplementary Materials

The following supporting information can be downloaded at: https://www.mdpi.com/article/10.3390/gels8030188/s1, Figure S1: SEM images of the GA-O-09/Cu^2+^ shrunken gel with (**a**) 0.1, (**b**) 0.3, (**c**) 0.4, (**d**) 0.6, (**e**) 0.7, and (**f**) 0.9 equivalent of Cu^2+^, respectively; Table S1: The MICs (μg/mL) of the GA-O-09/Cu^2+^ hydrogels with different equivalents of Cu^2+^^;^ Figure S2: Molecular structures of the studied dyes; Table S2. BET of (**a**) GA-O-09/Cu^2+^ and (**b**) GA-O-09 hydrogels; Figure S3: UV–vis spectra of the dye solutions with the GA-O-09 gel: (**a**) EY, (**b**) MO, (**c**) MB, (**d**) R6G, (**e**) MC, and (**f**) a mixture of MO/MB for the indicated time; Figure S4: Zeta potential of (**a**) GA-O-09/Cu^2+^ and (**b**) GA-O-09 hydrogels; Figure S5: FT-IR spectra of GA-O-09/Cu^2+^ gel-absorbed MC and MO, respectively; Figure S6: SEM images of the morphology of (**a**) GA-O-09/Cu^2+^-MO, (**b**) GA-O-09/Cu^2+^-MO; Figure S7: SEM images of the morphology of regeneration GA-O-09/Cu^2+^ hydrogel; Figure S8. FT-IR spectra of GA-O-09/Cu^2+^ and regeneration GA-O-09/Cu^2+^ hydrogel.

## References

[1] Rizzello L., Pompa P.P. (2014). Nanosilver-Based antibacterial drugs and devices: Mechanisms, methodological drawbacks, and guidelines. Chem. Soc. Rev..

[2] Yan L., Fukushima K., Coady D.J. (2013). Broad-Spectrum Antimicrobial and Biofilm-Disrupting Hydrogels: Stereocomplex-Driven Supramolecular Assemblies. Angew. Chem. Int. Ed..

[3] Zhou A.Y., Zhang Y.Y., Qu Q.L. (2021). Well-Defined multifunctional superhydrophobic green nanofiber membrane based-polyurethane with inherent antifouling, antiadhesive and photothermal bactericidal properties and its application in bacteria, living cells and zebra fish. Compos. Commun..

[4] Yang K., Han Q., Chen B., Zheng Y., Zhang K., Li Q., Wang J. (2018). Antimicrobial hydrogels: Promising materials for medical application. Int. J. Nanomed..

[5] Li Y., Rodrigues J., Tomás H. (2012). Injectable and biodegradable hydrogels: Gelation, biodegradation and biomedical applications, Chem. Soc. Rev..

[6] Wei D.W., Wei H., Gauthier A.C., Song J., Xiao H. (2020). Superhydrophobic modification of cellulose and cotton textiles: Methodologies and applications. J. Bioresour. Bioprod..

[7] Guo S.Z., Chen S.L., Cao N.N., Zheng W.D., Li D.L., Sheng Z.J., Xu X.T., Zhang Q.M., Zheng X., Wu K.K. (2021). A novel 18β-glycyrrhetinic acid derivative supramolecular self-assembly hydrogel with antibacterial activity. J. Mater. Sci..

[8] Guo S.Z., Fan Y.Q., Hong W.Q.D., Liang J.F., Luo Z.J., Zheng W.D., Li D.L., Gan L.S., Xu X.T., Wu R.H. (2021). Super-Rapid formation of a novel super-supramolecular hydrogel with excellent antimicrobial activity. Compos. Commun..

[9] Liu J., Fan Y.Q., Zhang Q.P., Yao H., Zhang Y.M., Wei T.B., Lin Q. (2019). Super metal hydrogels constructed from a simple tripodal gelator and rare earth metal ions and its application in highly selective and ultrasensitive detection of histidine. Soft Matter..

[10] Xiong R., Xu R.X., Huang C., Smedt S.D., Braeckmans K. (2021). Stimuli-Responsive nanobubbles for biomedical applications. Chem. Soc. Rev..

[11] Villanueva M.E., Diez A., González J.A., Pére C.J., Orrego M., Piehl L.L., Teves S.A., Copello G.J. (2016). Antimicrobial Activity of Starch Hydrogel Incorporated with Copper Nanoparticles. ACS Appl. Mater. Interfaces.

[12] Zanjanchi M.A., Golmojdeh H., Arvand M. (2009). Enhanced adsorptive and photocatalytic achievements in removal of methylene blue by incorporating tungstophosphoric acid-TiO_2_ into MCM-41. J. Hazard. Mater..

[13] Qin L., Duan P., Fan X., Li Z., Liu M. (2013). A metal ion triggered shrinkable supramolecular hydrogel and controlled release by an amphiphilic peptide dendron. Chem. Commun..

[14] Lian Z., Jiang M., Xing L.B. (2019). Artificial light-harvesting supramolecular assemblies with different morphology formed by cucurbit[n]urils-based host-guest complexation. J. Photochem. Photobiol. A Chem..

[15] Yang Y., Zhang Z.Q., Ding J.M., Wang S.M., Ju Y.C. (2011). Crystal structure of tetrapyridinecopper(II) bis(triiodide), [Cu(C_5_H_5_N)_4_][I_3_]_2_. Z. Krist.—New Cryst. Struct..

[16] Liu J.G., Yin F., Hu J., Ju Y. (2021). Cu^2+^-Triggered shrinkage of a natural betulin-derived supramolecular gel to fabricate moldable self-supporting gel. Mater. Chem. Front..

[17] Xie Y.Y., Zhang Y.W., Qin X.T., Liu L.P., Wahid F., Zhong C., Jia S.R. (2020). Structure-Dependent Antibacterial Activity of Amino Acid-Based Supramolecular Hydrogels. Colloids Surf. B Biointerfaces.

[18] Chang H.W., Lin Y.S., Tsai Y.D., Tsai M.L. (2012). Effects of chitosan characteristics on the physicochemical properties, antibacterial activity, and cytotoxicity of chitosan/2-glycerophosphate/nanosilver hydrogels. J. Appl. Polym. Sci..

[19] Adak A., Ghosh S., Gupta V., Ghosh S. (2019). Biocompatible Lipopeptide-Based Antibacterial Hydrogel. Biomacromolecules.

[20] Cheng N., Hu Q., Guo Y., Wang Y., Yu L. (2015). Efficient and Selective Removal of Dyes Using Imidazolium-Based Supramolecular Gels. ACS Appl. Mater. Interfaces.

[21] Guan X.W., Lin Q., Zhang Y.M. (2019). Pillar[5]arene-Based spongy supramolecular polymer gel and its properties in multi-responsiveness, dye sorption, ultrasensitive detection and separation of Fe^3+^. Soft Matter.

[22] Njuguna D.G., Schnherr H. (2021). Xanthan gum hydrogels as high-capacity adsorbents for dye removal. ACS Appl. Polym. Mater..

[23] Li Y., Zhang Y., Wang G., Li S., Han R., Wei W. (2018). Reed biochar supported hydroxyapatite nanocomposite: Characterization and reactivity for methylene blue removal from aqueous media. J. Mol. Liq..

[24] Dutta J.D., Thakur D., Bahadur D. (2015). SnO_2_ quantum dots decorated silica nanoparticles for fast removal of cationic dye (methylene blue) from wastewater. Chem. Eng. J..

[25] Abdel Ghafar H.H., Ali G.A., Fouad O.A., Makhlouf S.A. (2015). Enhancement of adsorption efficiency of methylene blue on Co_3_O_4_/SiO_2_ nanocomposite. Desalination Water Treat..

[26] Nada A.A., Bekheet M.F., Roualdes S. (2018). Functionalization of MCM-41 with titanium oxynitride deposited via PECVD for enhanced removal of methylene blue. J. Mol. Liq..

