# Supplementary material for: Novel Natural Glycyrrhetinic Acid-Derived Super Metal Gel and Its Highly Selective Dyes Removal
Source: Gels. 2022 Mar 19;8(3):188. doi: 10.3390/gels8030188 (PMC8950348; doi:10.3390/gels8030188)
Supplement: Supplementary file 1 [file gels-08-00188-s001.zip › gels-1619382-supplementary.pdf]

gels

Supplementary Materials

Novel Natural Glycyrrhetinic Acid-Derived Super Metal Gel and Its Highly Selective Dyes Removal

Shengzhu Guo [1,2,†], Kaize Su [1,2,†], Huiji Yang [3], Wende Zheng [1,2], Zhen Zhang [1,2], Song Ang [1,2,*], Kun Zhang [1,2,*] and Panpan Wu [1,2,*]

Materials and General Methods

Materials

All reagents were purchased from commercial suppliers of Adamas Reagent Ltd. (Shanghai, China), all of the other reagents were of analytical grade, and water used in this work was of ultrapure grade. Flash chromatography was carried out with silica gel (200-300 mesh) which was supplied by Inno-chem Co., Ltd. (Beijing, China). The antimicrobial activity was assayed by using a Multi-model Plate Reader (Infinite 200, TECAN, Guangzhou, China). The bacterial strains of *Staphylococcus aureus* (ATCC 6538), *Staphylococcus aureus* subsp. *aureus* (ATCC 29213), *Staphylococcus epidermidis* (ATCC 12228), and methicillin-resistant *Staphylococcus aureus* were obtained from Guangdong Culture Collection Center (Guangdong, People's Republic of China). All the strains were cultured in Mueller-Hinton broth (MHB).

Synthesis and Characterization of GA-O-09

Synthesis and characterization of GA-O-09 were carried out as previously reported.

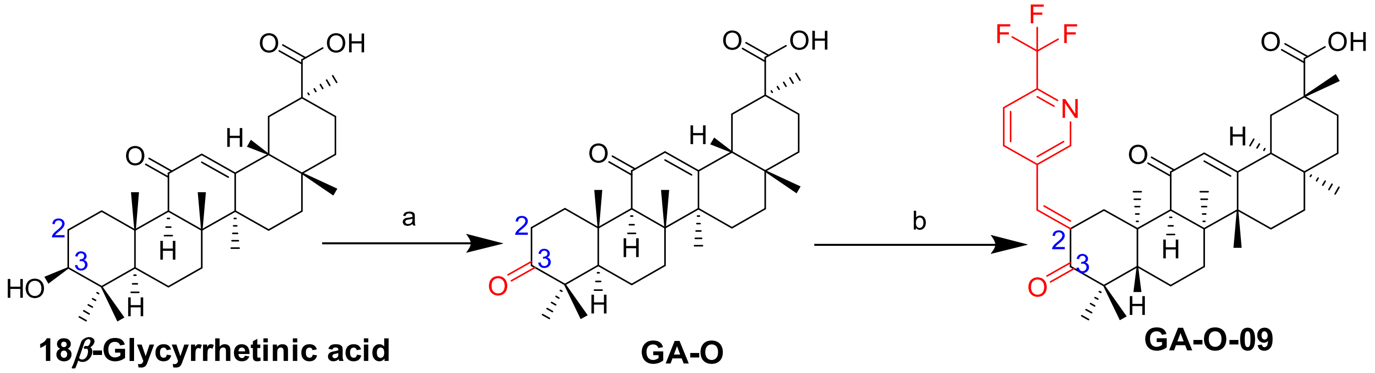

Scheme S1. Synthesis of 18β-glycyrrhetinic acid derivative GA-O-09. Reagents and conditions: a) ac-etone, Jones Reagents, 0 °C, 2 h, 90%; b) ethanol, KOH, 6-trifluoromethylpyridine-3-formaldehyde, r. t. (room temperature), 4 h, 76.8%.

Preparation of the GA-O-09/Cu^{2+} Hydrogel

15 mg of GA-O-09 was weighed out into a test bottle. Then, 0.52 mL ethanol was added into that test bottle followed by ultrasound, then 0.48 mL deionized water with copper sulfate was added into the mixture and the supramolecular hydrogel was constructed immediately.

Antibacterial Assays

The minimum inhibitory concentration (MIC) was determined by a microdilution method in 96-well plates according to Clinical and Laboratory Standards Institute (CLSI), with a slight modification. Each well received certain quality of xero-hydrogel, and 195

μL of MHB inoculated with the test microorganism (1.5 × 10^5 CFU/mL); Gatifloxacin was treated as positive control and DMSO was treated as negative control. The microplates were incubated in a bacteriological oven for 24 h at 37°C, and the susceptibility results of tested derivatives were monitored by measuring the absorbance at 600 nm using a Multimodel Plate Reader (Infinite 200).

Calculation of Shrinkage Ratio

The shrinkage ratio could be quantitatively calculated using eqn (1):

$$\text{Shrinkage ratio} = (m_o\text{-}m_s)/m_o \times 100\% \quad (1)$$

where m_s and m_o are the weight of the shrunken and original gels, respectively.

Assembly Mechanism

Fourier transform infrared (FT-IR) spectra were obtained using a Bruker spectrometer. Scanning electron microscopy (SEM) images were acquired using an SU-8010 instrument (the accelerating voltage was 10 kV). Samples were freeze-dried before measurement. UV-vis titration was recorded using a TU-1901 Spectrometer (1.0 cm quartz cuvette). GA-O-09 (70 mM) and copper nitrate (7 mM) were dissolved in $CH_3OH/CHCl_3$ (v/v, 1/2) to preclude the formation of precipitates or gel during the titration.

Dye Adsorption Experiment

15 mg of xerogel was soaked in the dye solution, shaken, and mixed for 5 minutes, and then allowed to stand. Then the suspension in the bottle (1.00 mL) was taken by a syringe at different intervals and then filtered immediately by using a LABMAX 0.2 μm membrane filter. UV–vis spectroscopy was used to determine the residual concentration of the pollutants in each sample.

Dye Adsorption Kinetics

In this work, two commonly used models were chosen to study the adsorption kinetics and mass transfer effects of different dyes. When membrane diffusion is rate-controlled (simple adsorption or physisorption), applying the pseudo-first-order rate equation, which is represented as follows.

For the rate constant for first order chemical sorption,

$$\frac{dq_t}{d_t} = k_1(q_{e-}q_t) \quad \text{(S1)}$$

Integarting this for the boundary conditions t=0 to t=t and q_t=0 to q_t=q_e, gives:

$$log(q_{e-}q_t) = \log(q_t) - \frac{k_1}{2.303}t \quad \text{(S2)}$$

Where k_1 is the rate constant in min^{-1}, t is real-time of adsorption in min, q_t is the adsorption capacity at the time of t, and q_e is the adsorption capacity at equilibrium, with the unit of $mg{\cdot}g^{-1}$, respectively.

For the rate constant for first order chemical sorption,

$$\frac{dq_t}{d_t} = k_2(q_{e-}q_t)^2 \quad \text{(S3)}$$

Integarting this for the boundary conditions t=0 to t=t and q_t=0 to q_t=q_e, gives:

$$\frac{1}{q_e - q_t} = \frac{1}{q_e} + k_2 t \quad \text{(S4)}$$

(S4) can be rearranged to obtain a linear form of

$$\frac{t}{q_t} = \frac{1}{k_2 {q_2}^2} + \frac{1}{q_2} t \quad (S5)$$

where the k_2 is the rate constant in ($mg \cdot g^{-1} \cdot min^{-0.5}$).

Adsorption Thermodynamics

The adsorption Gibbs free energy change (ΔG^0, $kJ \cdot mol^{-1}$), the adsorption enthalpy change (ΔH^0, $kJ \cdot mol^{-1}$), and the adsorption entropy change (ΔS^0, $J \cdot mol^{-1} \cdot K^{-1}$) were calculated according the following formula.

$$\Delta G^0 = -RT ln K_d \quad (S6)$$

$$\ln K_d = \frac{\Delta G^0}{R} - \frac{\Delta H^0}{RT} \quad (S7)$$

Where R = 8.314 $J \cdot mol^{-1} \cdot K^{-1}$, T is absolute temperature (K), and K_d (L/g) is the distribution coefficient of adsorbent that equals to q_e/C_e.

Results and Discussion

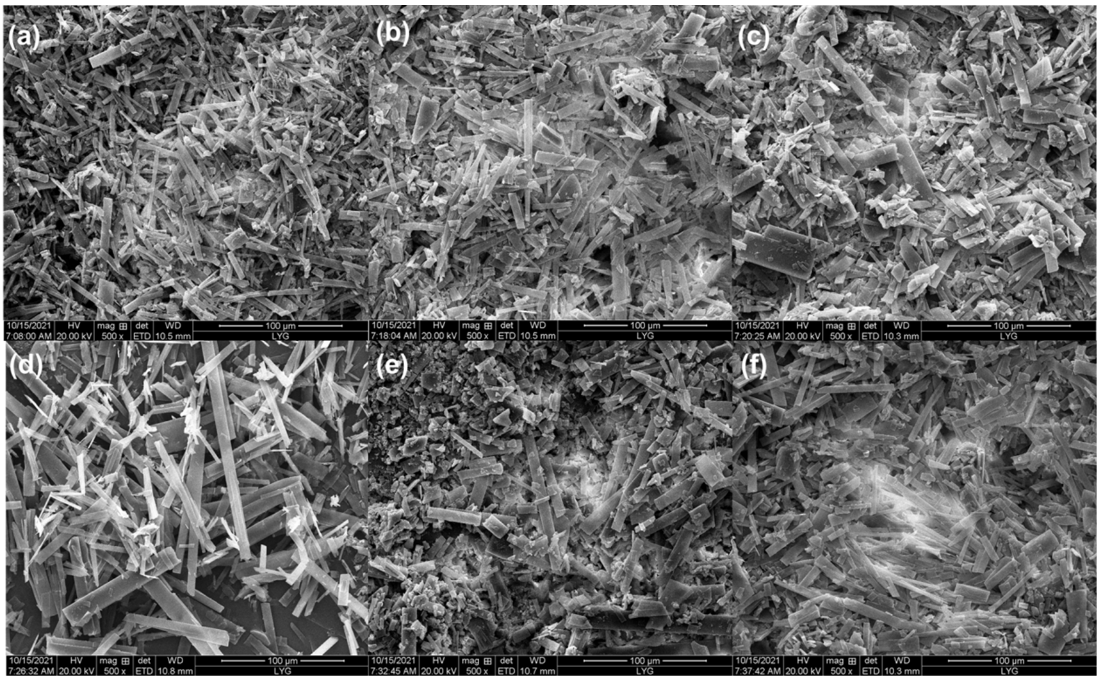

Figure S1. SEM images of the GA-O-09/Cu^{2+} shrunken gel with (**a**) 0.1, (**b**) 0.3, (**c**) 0.4, (**d**) 0.6, (**e**) 0.7, and (**f**) 0.9 equivalent of Cu^{2+}, respectively.

Table S1. The MICs (μg/mL) of the GA-O-09/Cu^{2+} hydrogels with different equivalents of Cu^{2+}.

**Sample**	**MICs of selected bacteria (μg/mL)**			
	Staphylococcus aureus **(ATCC 6538)**	*Staphylococcus aureus* **subsp.** *aureus* **(ATCC 29213)**	*Staphylococcus epidermidis* **(ATCC 12228)**	*Methicillin-resistant Staphylococcus aureus* **(MRSA)**
0 equiv.	2.5	2.5	2.5	5
0.1 equiv.	2.5	2.5	2.5	5
0.2 equiv.	2.5	2.5	2.5	5
0.3 equiv.	2.5	2.5	2.5	5
0.4 equiv.	2.5	2.5	2.5	5
0.5 equiv.	2.5	2.5	2.5	5
0.6 equiv.	2.5	2.5	2.5	5

0.7 equiv.	2.5	2.5	2.5	5
0.8 equiv.	2.5	2.5	2.5	5
0.9 equiv.	2.5	2.5	2.5	5
1.0 equiv.	2.5	2.5	2.5	5
$CuSO_4$	NT	NT	NT	NT

NT, not tested.

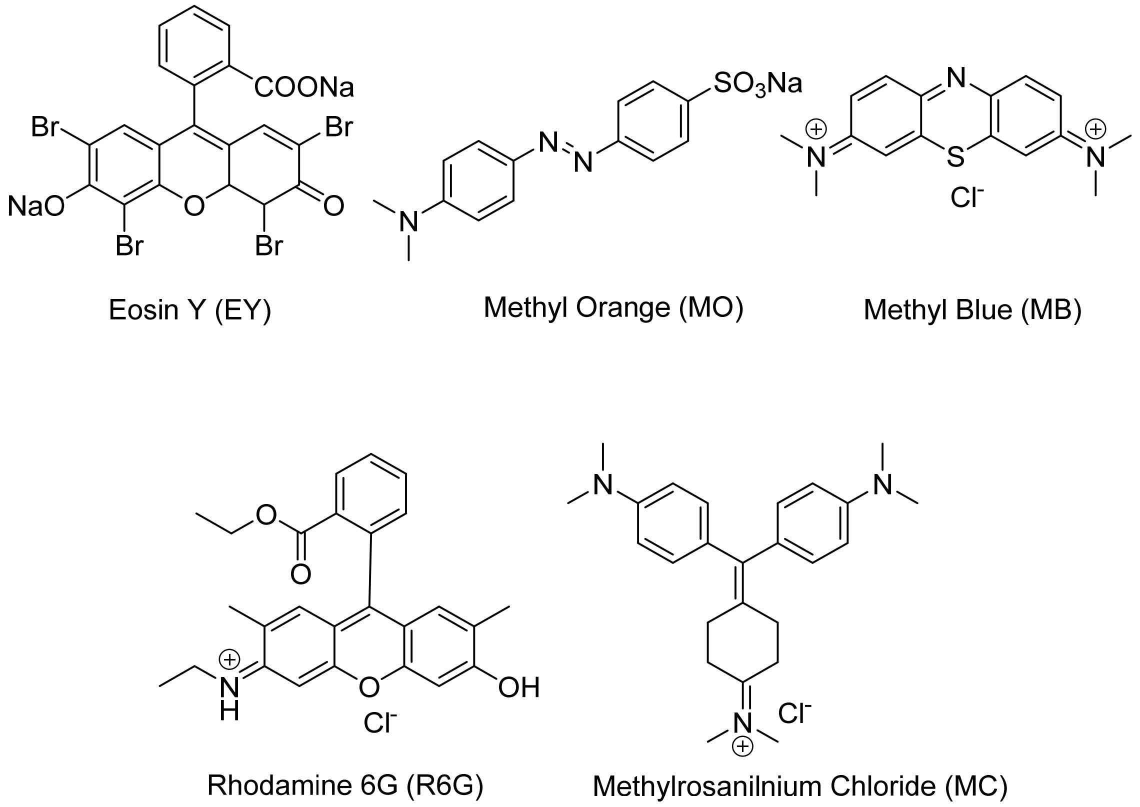

Figure S2. Molecular structures of the studied dyes.

Table S2. BET of (a) GA-O-09/Cu^{2+} and (b) GA-O-09 hydrogels.

	**GA-O-09/Cu^{2+}**	**GA-O-09**
BET Surface Area	8 m^2/g	15 m^2/g
Langmuir Surface Area:	141.3094 m^2/g	10.2995 m^2/g
Adsorption average pore diameter (4V/A by BET):	15.8193 nm	15.4044 nm
Desorption average pore diameter (4V/A by BET):	15.8193 nm	15.4044 nm

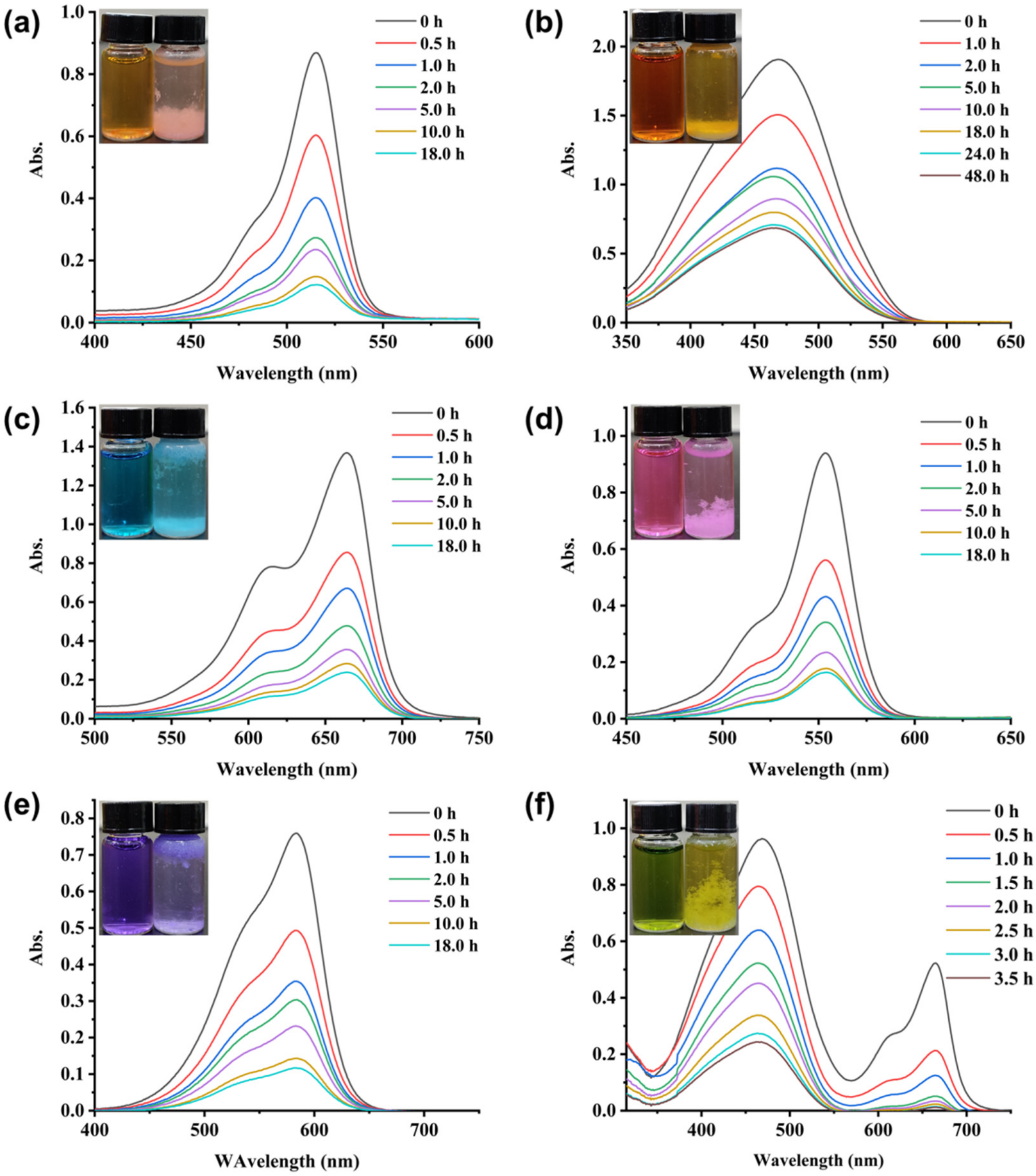

Figure S3. UV-vis spectra of the dye solutions suspended with the GA-O-09 gel: EY (**a**), MO (**b**), MB (**c**), R6G (**d**), MC (**e**), and a mixture of MO/MB (**f**) for the indicated time.

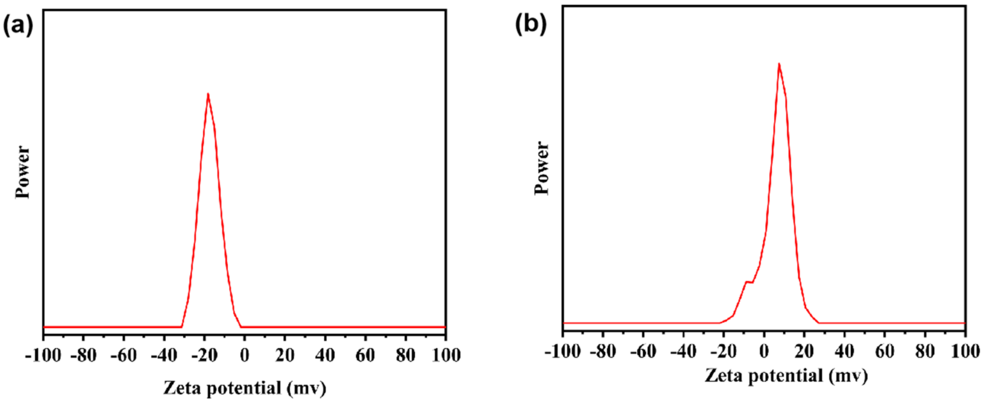

Figure S4. Zeta potential of (**a**) GA-O-09/Cu^{2+} and (**b**) GA-O-09 hydrogels.

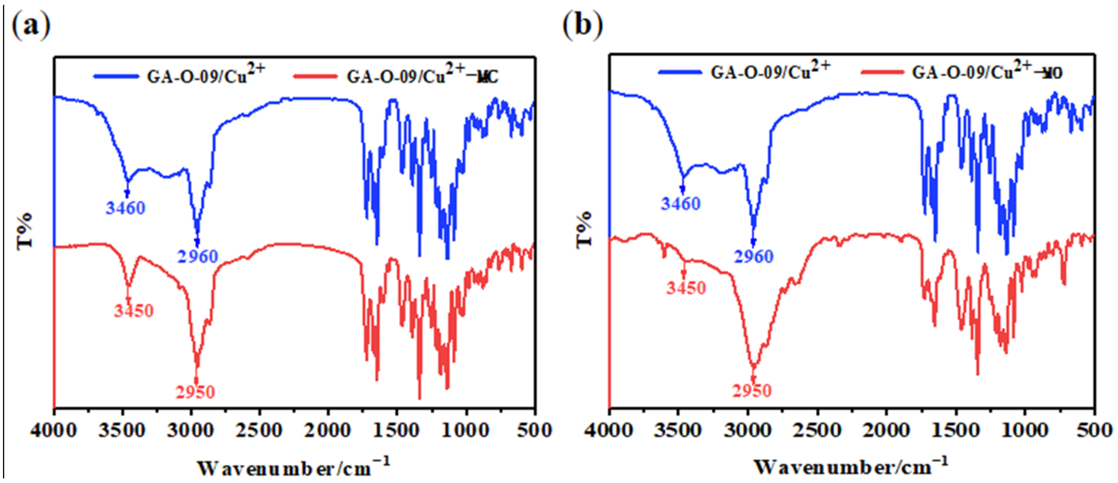

Figure S5. FT-IR spectra of GA-O-09/Cu^{2+} gel absorbed (**a**) MC and (**b**) MO, respectively.

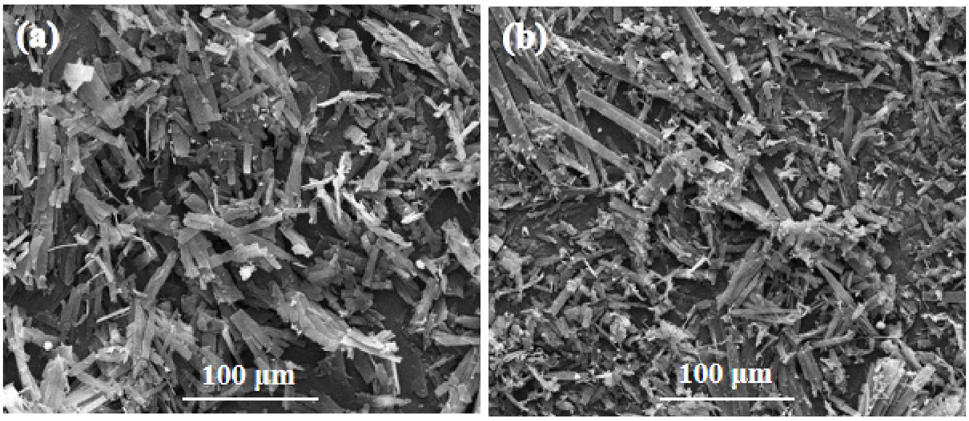

Figure S6. SEM images of the morphology of (**a**) GA-O-09/Cu^{2+}-MO, (**b**) GA-O-09/Cu^{2+}-MO.

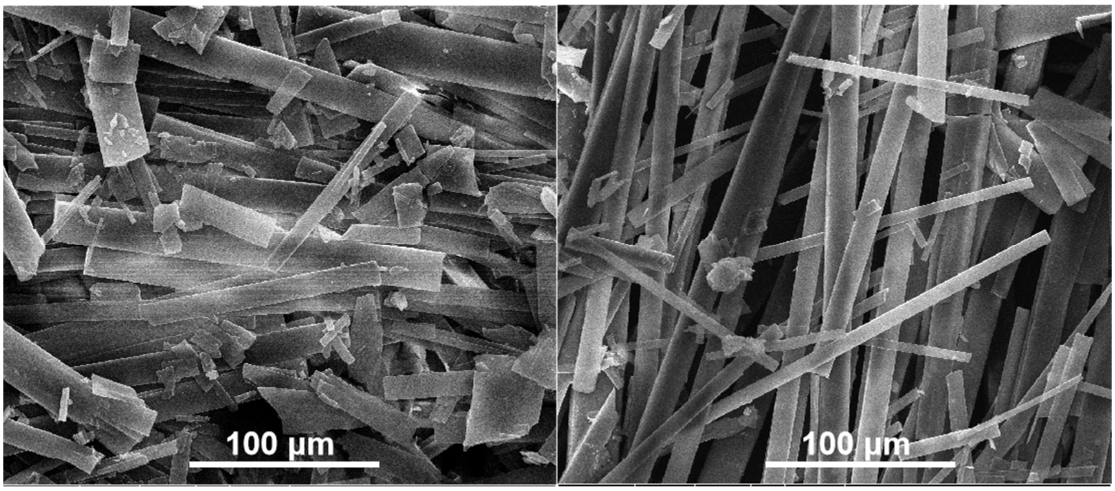

Figure S7. SEM images of different areas of the morphology from regeneration GA-O-09/Cu^{2+} hydrogel.

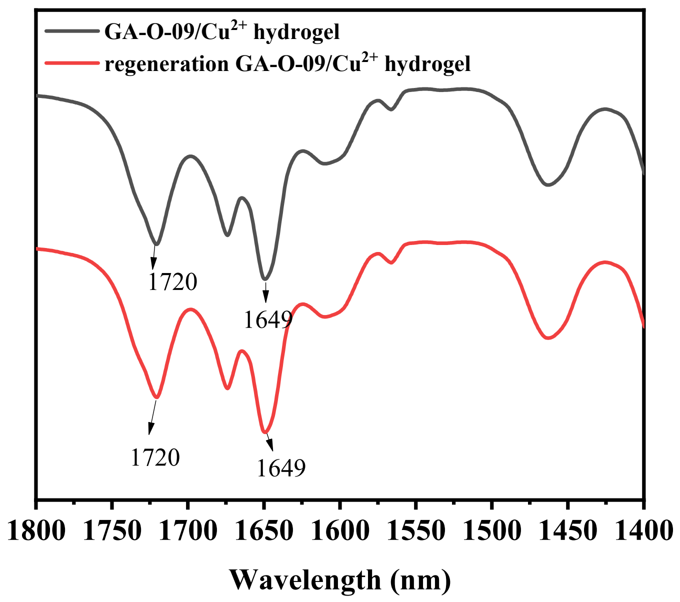

Figure S8. FT-IR spectra of GA-O-09/Cu^{2+} and regeneration GA-O-09/Cu^{2+} hydrogel.
